# The application effectiveness of the “One Case to the End” teaching model based on BOPPPS in pediatric nursing: a quasi-experimental study

**DOI:** 10.1186/s12909-025-07073-2

**Published:** 2025-04-17

**Authors:** Xuejun Xiao, Ying Xie, Xiangfeng Zhao, Xiaohong Dai, Feng Liang, Wanchao Wang, Xianhua Zhang

**Affiliations:** 1 Department of Gynecological and Pediatric Nursing, College of Nursing, Guilin Medical University, Guilin, 541001 China; 2 Department of Immunology, College of Basic Medical Sciences, Guilin Medical University, Guilin, 541001 China; 3 Department of Obstetrics, The First Affiliated Hospital of Guilin Medical University, Guilin, 541001 China; 4 Thyroid Hernia Pediatric Surgery,The Second Peoples Hospital of Foshan, Foshan, 528000 China

**Keywords:** BOPPPS, One Case to the End, Professional Values, Critical Thinking, Pediatric Nursing

## Abstract

**Background/aim:**

The traditional teacher-centered teaching method of pediatric nursing only focuses on the imparting of knowledge, ignoring the cultivation of students’ autonomous learning abilities and the establishment of critical thinking skills. Moreover, the humanized care for pediatric patients is merely a formality, which can’t make a deep impression. This study aimed to explore the application effectiveness of the “One Case to the End” teaching model based on BOPPPS in pediatric nursing teaching.

**Materials and methods:**

A quasi-experimental research design and convenience sampling were conducted in this study. A total of 276 nursing undergraduates from two parallel classes of a nursing college were recruited, with 138 students in the Grade 2020 serving as the control group and receiving the traditional teaching method, while the students of Grade 2021 served as the experimental group and implemented the “One Case to the End” teaching model based on BOPPPS. At the end of the semester, the study compared the two groups of students’ academic performance, the level of nurses’ professional values and critical thinking abilities. Additionally, it surveyed the experimental group’s satisfaction with the curriculum teaching reform.

**Results:**

The academic performance, the score of nurses’ professional values and the score of critical thinking abilities of the experimental group were significantly higher than those of the control group (all *P* < *0.01*), and the students in the experimental group had a relatively high evaluation of the reform satisfaction with the new teaching model.

**Conclusion:**

The “One Case to the End” teaching model based on BOPPPS can effectively enhance students’ academic performance, nurses’ professional values and critical thinking abilities, and improve the teaching quality of pediatric nursing.

**Supplementary Information:**

The online version contains supplementary material available at 10.1186/s12909-025-07073-2.

## Introduction

Pediatric Nursing is a compulsory and core course for undergraduate nursing students, bearing the honorable mission of promoting the normal growth and development of children as well as their physical and mental health [1]. However, due to the special characteristics of the anatomy, physiology and psychological development of the objects studied in this course, coupled with the differences in organ function, immune and response ability to disease, emotional reaction patterns, communication and expression skills among children of various age stages [2, 3], pediatric patients tend to have relatively poor safety awareness and cooperativeness during treatment [4], leading to noticeably greater stress on pediatric nursing compared to adult nursing [5]. This situation may result in family members' dissatisfaction with the nursing work [6]. Compared to other clinical nursing specialties, the teaching of pediatric nursing should place even greater emphasis on the cultivation of students' clinical practical abilities [7], the investment in emotional values [8], and the establishment of clinical judgment and thinking skills [9]. Pediatric nurses, as advocates for child patients, should have high professional values and keen critical thinking abilities in order to protect the lives and health of their patients [10, 11].

The current undergraduate nursing curriculum for Pediatric Nursing predominantly employs traditional lecture-based teaching methods, i.e., teacher-centered and didactic teaching methods [12], in which the teacher primarily focuses on imparting knowledge and does not emphasize the application of knowledge, leaving students in a passive role as mere recipients of information [13, 14]. Although this method can deliver a great amount of teaching contents, it risks disconnecting theoretical learning from clinical practice [15]. It also fails to effectively cultivate students' self-directed learning abilities [16], enthusiasm for learning [17], teamwork skills and critical thinking capabilities [18], resulting in suboptimal learning outcomes for students [19]. Furthermore, the teacher-centered didactic teaching method deprive students of the ability to actively explore knowledge. Coupled with the particularities of pediatric patients and the increasing awareness of rights protection among parents [7], this leads to a deficiency in students' perceptual abilities and empathy towards pediatric patients and their parents [20]. Consequently, it further increases the difficulty for students to engage in clinical practice of pediatric nursing [11], thereby affecting the achievement of undergraduate nursing education objectives [21]. Therefore, there is an urgent need to innovate teaching strategies for pediatric nursing education and establish a student-centered teaching philosophy to effectively enhance students' professional values and critical thinking skills.

The BOPPPS teaching model consists of six components: Bridge-in (B), Objective/Outcome (O), Pre-assessment (P), Participatory Learning (P), Post-assessment (P), and Summary (S) [22]. It is a novel teaching paradigm oriented by educational goals and centered on students [23]. It enables students to effectively integrate into classroom teaching, fully exert their initiative and creative thinking, and provide real-time feedback on teaching situations, thereby cultivating the establishment of students' critical thinking abilities and enhancing the effectiveness of classroom teaching [24, 25]. This teaching model has been widely used in medical education, such as theoretical and experimental teaching, clinical practice or pre-service training for newly recruited health care workers, all of which have achieved satisfactory teaching results [26, 27]. The teaching model based on BOPPPS can mobilize students' enthusiasm for learning, stimulate their self-learning ability, and improve teaching effectiveness and trainees' satisfaction with the instructors and teaching methods [28], even demonstrating its irreplaceable superior potential compared with the traditional teaching model [29]. The BOPPPS model, as an open-ended instructional design model, will have a more positive impact on teaching effectiveness and students' attitudes toward learning when combined with other learning strategies [15].

The “One Case to the End” teaching method is a kind of case teaching method. It involves teachers selecting a typical case that runs through the entire course content based on students' cognitive patterns and curriculum requirements, all centered around a specific theme [30]. Teachers set up questions with varying levels of difficulty according to the teaching needs, introduce real-case scenarios to create clinical contexts, and concretize abstract theoretical knowledge through case study activities, so that students can learn and apply knowledge in real situations. This contextualized learning approach not only increases the interest and realism of learning, but also helps to cultivate students' compassion and empathy [31], form critical thinking and professional value identification [32], and enhance practical skills [33]. Through activities such as role-playing and interactive discussions, students reflect on their learning process, identify problems and deficiencies, and continuously adjust and optimize their cognitive structures. This helps them better understand and master knowledge, which is of great significance for improving the quality of course teaching and enhancing teaching effectiveness [34].

Both BOPPPS model and the “One Case to the End” teaching method are grounded in constructivist learning theory [33, 35],whose core philosophy emphasizes “Student-centeredness, fully leveraging students’ subjective initiative, and cultivating their proactivity and consciousness in exploring, discovering, and constructing knowledge.” The BOPPPS teaching model and the “One Case to the End” pedagogy have been widely applied across disciplines including medicine, natural sciences, high school geography, and ideological-political education, demonstrating positive teaching outcomes [24, 26, 30, 31, 36]. While both the BOPPPS teaching model and the “One Case to the End” pedagogy have distinct advantages, they also exhibit certain limitations. The BOPPPS teaching model decomposes classroom instruction into reasonable components, forming a complete process-oriented instruction. However, it does not specify who should guide each stage or how to implement effective measures within these segments [37]. The successful implementation of the “One Case to the End” teaching method relies heavily on effective guidance from teachers. Teachers play the role of guide and facilitator throughout the entire teaching process, responsible for steering the direction of students' learning, stimulating their interest in learning, and helping students to deeply understand and mastery of knowledge [33]. Therefore, an integrated application of these two pedagogical approaches can effectively leverage their complementary strengths. Currently, the integration of BOPPPS teaching model and the “One Case to the End” method has rarely been applied in pediatric nursing courses. In this study, we explored the integration of “One Case to the End” into the BOPPPS teaching model in the theory and laboratory teaching of pediatric nursing, with the aim of investigating the impact of this new teaching strategy on the learning outcomes of undergraduate nursing students, including students’ exam scores, professional values and critical thinking abilities, as well as the evaluation of satisfaction of this new teaching reform.

## Method

### Design and participants

A quasi-experimental research design was conducted in this study. A convenience sampling method was used. Participants in this study were second-year undergraduate nursing students enrolled in a four-year undergraduate nursing training program at a medical university in Guilin City, Guangxi Zhuang Autonomous Region, China. Inclusion criteria were as follows: (1)Full-time undergraduate nursing students currently enrolled in school; (2)No current physical or mental health conditions or symptoms; (3)No prior experience with BOPPPS teaching method or the “One Case to the End” methodology; (4)Willingness to participate fully and complete all survey questionnaires voluntarily, along with signed informed consent. Previous experience with BOPPPS or the “One Case to the End” learning method, inability to complete the survey questionnaires diligently, and those who leave or dropping out were considered exclusion criteria. A total of 138 students from the Grade 2020 were selected as the control group, implementing traditional teaching methods, while 138 students from the Grade 2021 were selected as the experimental group, adopting the “One Case to the End” teaching model based on BOPPPS. All participating classes were parallel classes, and the same teaching strategies were applied consistently across all classes in the control group to ensure uniformity and comparability.

Pediatric Nursing course follows a progressive logic of “from health to disease, from mild to severe, and from simple to comprehensive”, forming three progressively advancing teaching modules: “health protection—disease care—critical care”. The course consists of 63 credit hours in total. Specifically, there are 39 h of theoretical classes, 18 h of practical experiments, and 6 h of guided study. Both groups began this course in their second year, after completing the foundational medical courses and basic nursing studies. See Table 1 for more information on the course. Both groups were taught by the same teachers and used the seventh edition of “Pediatric Nursing” [1] published by People’s Medical Publishing House, China. The teaching syllabus followed the 2020 version of the student training program of the school. Additionally, characteristics (including age, gender, and pre-course GPA), professional values, and critical thinking abilities were assessed prior to the experiment for both groups of students, and results showed that the two groups were comparable. Data were also collected in December 2022 and December 2023 at the end of the semester. The study was approved by the Medical Ethics Committee of Guilin Medical University (No. GLMC20210505), and informed consent was obtained from each participant.

**Table 1:** Details of the pediatric nursing course

Item	Description
Course Title	Pediatric Nursing
Course Duration	18 weeks
Total Number of Credit Hours	63 credit hours (theoretical classes: 39, practical experiments: 18, guided study:6)
Class Schedule	**Frequency:** once a week (theoretical class); once every two weeks (practical experiments and guided study) **Duration:** 40 min per class
Number of Instructors	6 nursing school full-time teachers; 6 clinical pediatric and neonatal nursing instructors
Course Content	**Theoretical contents:** Principles of Child Growth and Development, Child Health Care, Diseases and Nursing Care of Various Systems, and Critical and Emergency Care **Practical Experiments contents:** Common Neonatal Nursing Techniques, Common Pediatric Nursing Techniques, Pediatric Venipuncture, Neonatal Ward Practicum, Pediatric Ward Practicum, Scenario-Based Case Drills **Guided Study contents:** Case nursing documentation and presentation, Online Self-Study
Supervised Clinical Training	**Hours:** 6 h **Setting:** 3 h in pediatric wards and neonatal intensive units respectively **Supervision:** 1 instructor for every 5 students **Objectives:** Observe and participate in real healthcare environments to strengthen the integration of theory and practice; learn and practice various clinical skills to enhance hands-on abilities; learn to communicate with patients and their families, foster professional attitudes and behaviors; solve practical problems under the guidance of instructors to improve capabilities in handling emergencies and complex cases

### Implementation of teaching method

#### Control group

The control group adopted traditional teaching methods, which the teachers imparted knowledge and students passively learned and received it. Before class, teachers used Rain Classroom [38] to release relevant course materials for students to preview and watched the “Pediatric Nursing” course from the Chinese University MOOC [39] independently, and completed post-class questions. Theoretical classes were primarily taught by teachers, combining teaching methods such as Problem-Based Learning (PBL), Case-Based Learning (CBL), and case discussions to explain theoretical knowledge, while using Rain Classroom to send questions to test students’ learning outcomes in class. After class, related chapter assignments were given, such as finishing case exercises or drawing mind maps. In the lab sessions, firstly teachers demonstrated related experimental operations, showed the experimental process, emphasized important considerations, and guided students to watch operation videos. Then, students were arranged to practice in groups, with teachers providing guidance from the side. Ten minutes before class, students were invited to show the operations, meanwhile the teacher gave comments and feedback, as well as summarized the key points, difficulties, and precautions of this experiment.

#### Experimental group

Experimental group implemented the “One Case to the End” teaching model based on BOPPPS for both theoretical and experimental teaching.

#### Preparation work

##### The construction of a clinical case library

The construction of a clinical case library involved the course supervisor contacting pediatric nursing clinical teachers to collect clinical cases of typical diseases in the NICU and pediatric wards. The requirement was to have common diseases in the past three years, with at least 2 to 4 typical common and frequently occurring diseases for each system, and complete clinical datas. When designing the clinical case problems, it was important to highlight the connection between the case and the teaching objectives, such as deducing the etiology of the disease based on the children’s illness process, and inferring the auxiliary examination methods for the disease from the physical examination results. The course coordinator reviewed the completeness and rationality of the cases, supplemented relevant materials, and categorizes the case library into three major teaching modules: Home Health Care and Nursing for Children, Nursing Care for Common Pediatric Diseases, and Nursing for Pediatric Critical Illnesses. Over 30 clinical cases have been collected. One clinical case from each of the three modules was selected for demonstration (see Table 2 for details). To ensure consistency in teaching content, the same cases were used for both theoretical and practical instruction to guarantee coherence of knowledge.

**Table 2:** Clinical cases from three major modules (Partial)

Module 1 Home Health Care and Nursing for Children	Children’s Growth and Healthcare Xiaoming, male, 1 month old, was taken to the community hospital for a physical examination by his parents. The nurse conducted measurements of physical growth and development, which showed a weighed of 8.5 kg, length of 60 cm, head circumference of 40 cm, and chest circumference of 39 cm. While changing the diaper, the nurse noticed pus-like yellow fluid oozing from the umbilical area. Xiaoming’ s parents lacked knowledge on how to safely bathe their baby, change diapers, and perform newborn massage. The nurse is also prepared to administer vaccines to Xiaoming Questions for Theoretical Class: (1)What laws does the physical growth and development of children follow? (2)What are the commonly used indicators for physical growth and development? what are their normal values, and what do these values signify? (3)What does the pus-like yellow fluid oozing from Xiaoming’ s umbilical area indicate? How should it be handled? (4)What vaccines should be administered to Xiaoming at his current age? What are the common adverse reactions to these vaccines? Questions for Laboratory Class: (1)Are Xiaoming’ s physical examination results indicators normal? (2)Please perform the correct measurement of physical growth and development for Xiaoming (3)Please address the pus-like yellow fluid oozing from Xiaoming’ s umbilical area (4)Please demonstrate to Xiaoming’ s parents the procedures for bathing the baby, changing diapers, and performing newborn massage (5)Please administer the necessary vaccines to Xiaoming
Module 2 Nursing Care for Common Pediatric Diseases	Nursing Care for Children with Malnutrition Lily, female, 2 years old Chief Complaint: refusal to eat accompanied by weight loss for 1 year History of Present Illness: One year prior to admission, the child experienced recurrent diarrhea, poor appetite, with daily milk intake less than 200 ml and gradually lagging growth and development. The patient also exhibited delayed motor function, lethargy, and restless sleep Physical Examination: Temperature (T) 36 °C, Heart Rate (HR) 95 beats per minute, Respiratory Rate (R) 35 breaths per minute, Weight 8 kg, Height 84 cm. The patient appeared lethargic with pale complexion, dry skin, dull and yellow hair, and significant weight loss. The head circumference was normal. The pharynx was normal (-), and the lung sounds were clear bilaterally. The heart rate was 95 beats per minute, with a regular rhythm and no pathological murmurs. The abdomen was soft, with the liver palpable 2 cm below the costal margin and 1 cm below the xiphoid process, with a moderate consistency. The spleen was not palpable. Subcutaneous fat was almost completely absent, and there was no edema. The muscle tone of the limbs was low, with normal physiological reflexes and no elicited pathological reflexes Laboratory Tests: Complete blood count (CBC) showed hemoglobin (Hb) at 92 g/L, other parameters were within normal range. Urinalysis, stool examination, and liver function tests were normal. Serum iron and zinc levels were below reference values. Chest X-ray did not reveal any abnormalities Diagnosis: Marasmus (wasting type of malnutrition) Questions for Theoretical Class: (1)What is malnutrition, and how many clinical types can it be classified into? (2)To what extent does this Lily suffer from malnutrition, and what evidence supports your judgment? (3)What are the causes of malnutrition among children in China, and what is the primary cause for this Lily’ s malnutrition? (4)What are the clinical manifestations of this Lily’ s malnutrition, and in what order does fat reduction occur? Which complication is most lethal? (5)What abnormal values do you see in the Lily’ s auxiliary tests, and what do they indicate? (6)What are the current nursing issues for Lily? How should targeted dietary care measures be developed for the existing nutritional problems? Questions for Laboratory Class: (1)Assuming the parents bring the child to the pediatric health clinic for a physical examination, please conduct a scenario role-play in groups to demonstrate the process and content of the physical examination (2)Lily’ s parents have questions about the introduction of complementary foods. Please explain in simple terms and understandable language the types of complementary foods suitable for this age group, as well as methods for preparing and combining these foods (3)Currently, the child is unable to eat orally, and the doctor has prescribed nasogastric feeding with milk. Please demonstrate the correct method of preparing the milk to the parents and perform the operation of inserting a nasogastric tube (4)Now that the child’ s condition has improved and they can eat by mouth, please instruct the parents on the correct bottle feeding method and emphasize the precautions
Module 3 Nursing for Pediatric Critical Illnesses	Nursing Care for Children with Convulsions Hanhan, male,1 year and 3 months old Chief Complaint: Cough with phlegm for 2 days, fever for 1 day, and one episode of seizure (as reported by parents) History of Present Illness: The child was brought to the hospital by his parents due to the aforementioned symptoms. While waiting for approximately 20 min in the outpatient area, the child suddenly became unconscious, stared upwards with both eyes, had cyanotic lips, clenched teeth, and convulsions in all four limbs. He was immediately transferred to the emergency room for resuscitation. One day prior to admission, the child developed a fever without any obvious cause. Six hours before admission, when his temperature peaked at around 38.8 °C, he experienced one episode of seizure characterized by upward rolling eyes, clenched teeth, and loss of consciousness, but no incontinence of urine or stool. This episode resolved spontaneously after about one minute. After resolution, he regained consciousness but appeared slightly weak, without sore throat, cough, headache, or vomiting. At the age of 6 months, the child had a similar episode associated with fever. There is no family history of seizures or epilepsy Physical Examination: Temperature (T) 39 °C, Heart Rate (HR) 142 beats per minute, Respiratory Rate (R) 40 breaths per minute. Examination revealed hyperemia of the pharynx, bilateral tonsils were mildly enlarged and congested (Grade I), without purulent discharge. Meningeal irritation signs were negative. Cardiovascular and pulmonary examinations were normal. Abdomen was soft and flat Laboratory Tests: White Blood Cell (WBC) count 15.66 × 10^9/L, Neutrophils (N) 76.3% Preliminary Diagnosis: 1. Febrile Seizure (Simple Type); 2. Acute Tonsillitis Questions for Theoretical Class: (1)What is a convulsion, and how does it differ from epilepsy? (2)What are the causes of Hanhan's convulsion, and why are children more prone to convulsion during children? (3)Which symptoms of a convulsion can help you quickly diagnose the condition, and what are the typical clinical manifestations? (4)Considering the current condition, which auxiliary examination do you think is most important to perform? (5)How would you provide first aid if you find Hanhan experiencing another seizure? (6)What are the main nursing issues currently facing Hanhan, and what corresponding nursing measures should be taken? (7)Discharge Health Education Poster for Hanhan and Family Questions for Laboratory Class: (1)Hanhan is currently having a seizure. Please conduct a scenario-based simulation in groups and implement the correct emergency measures for him (2)The child’ s seizure has stopped, and the nurse needs to understand the current body temperature situation. Please measure his vital signs (3)Doctor’ s order: 0.9% Saline 100 ml + Cefotaxime 0.35 g, intravenous infusion, once a day (Cefotaxime specification: 0.75 g/vial). Please prepare the medication solution and perform the scalp vein infusion according to the doctor’ s order (4)Hanhan currently has a body temperature of 38.5 °C. Please implement nursing measures of high fever for him (5)To enhance the awareness of convulsion prevention for Hanhan and his parents at home, please create an educational video on convulsion in children as a group project

##### Establishment and training of the teacher team

We established a teaching team comprising six full-time college teachers and six clinical teachers. The course supervisor assigned tasks to team members based on their professional strengths, pairing full-time teachers with clinical teachers for mutual support. By establishing a “Pediatric Nursing Teaching Faculty Discussion QQ Group”, online meetings were held every two weeks to discuss issues encountered during the teaching process, review and revise new clinical cases, and plan future teaching activities. This practice promoted knowledge sharing among teachers and facilitated timely adjustments to the teaching plan.

The course supervisor participated in the “Instructional Skills Workshop (ISW)” which was hosted by the School’s Center for Faculty Development and obtained a training qualification certificate for the BOPPPS teaching method. At the beginning of the semester, during the pre-semester collective preparation for “Pediatric Nursing”, the course supervisor demonstrated and trained the teachers who involved in pediatric theory and experimental teaching on the BOPPPS teaching method. The course supervisor provided the teaching faculty with a detailed explanation of how to effectively integrate the BOPPPS steps into the teaching process and the content of the course. They discussed the considerations to keep in mind when setting up each part of the content and conducted a demonstration lesson on “Child Nutrition” as an example. In addition, the clinical case libraries, question banks, courseware, and lesson plans related to the teaching content were uploaded to the “Pediatric Nursing Teaching Faculty Discussion QQ Group” files. This ensured that teachers can promptly obtain and share the latest teaching resources within the group, enriching classroom teaching content and activitie

##### Evaluation system

It primarily focused on teachers’ assessments, which mainly contained the evaluation of the students’ academic achievements, levels of professional values and critical thinking abilities in both groups. Additionally, it surveyed the experimental group’s satisfaction with the curriculum reform.

#### Implementation process

##### Before class

One week before the class, the teacher published study tasks through the class QQ group, uploaded relevant experimental operation videos, pushed preview courseware and clinical cases from Rain Classroom [38]. They also recommended online learning platforms and materials [39], requiring students to complete the pre-class preview tasks as required, finish role assignments and case rehearsals for role-playing scenarios.

## During class

1. Bridge-in The teacher used the Rain Classroom platform to deliver lectures, importing videos and clinical cases related to the teaching contents, posing questions to inspire students’ thinking, and arousing their interests in learning, thus to introduce the topic of the lecture.
2. Objective/Outcome The teacher briefly stated the learning objectives, letting students to clear about the learning goals and the lecture topic.
3. Pre-assessment The teacher pushed out pre-test questions based on the case, such as “What is the clinical diagnosis of this disease? What are the causes of this disease? How to formulate nursing measures for the patient?” etc., to test the students’ effectiveness of preview.
4. Participatory Learning Teachers provided targeted content instruction based on students’ pre-test performance combined with case studies. According to the development process of cases, treatment, and nursing experiences, teachers integrated relevant knowledge points and encouraged student participation in classroom activities. For instance, using videos, images, and models related to diseases to guide students in analyzing clinical manifestations and the significance of abnormal test results; promoting students’ ability to explore and solve problems through group discussions and sharing as well as in-class exercises; strengthening students’ moral integrity and professional confidence by citing outstanding examples within the industry; enhancing practical skills and teamwork spirit by having students work in groups to perform role-plays of case scenarios, thereby recreating the progression of cases and reconstructing theoretical knowledge.
5. Post-Assessment After the theoretical knowledge instruction was completed, the teacher set questions based on the outcome or prognosis of the case, such as “Family Care for the Child Patient” and “Health Education for the Child Patient at Home” to promptly assess students’ understanding and mastery of the course content.
6. Summary Finally, the teacher summarized the key points and difficulties with concise and clear language, making it easier for students to review and consolidate the knowledge after class, and inspiring them to continue thinking about the material beyond the classroom.

## After class

**Fig. 1:**
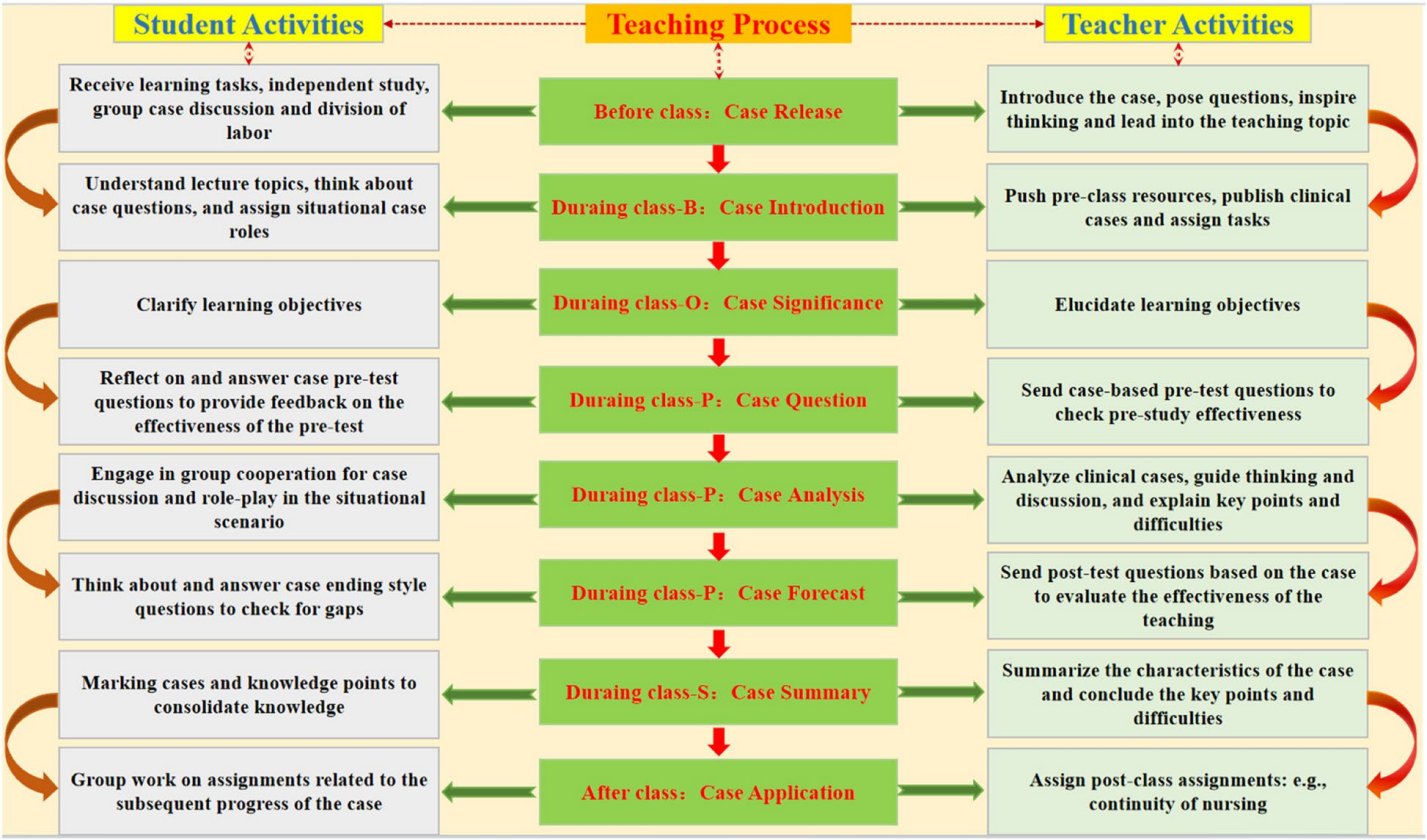
“One Case to the End” Teaching Model Based on BOPPPS in Pediatric Nursing

To extend and expand on the case studies, the teacher assigned homework in the class QQ group, such as “Create home care promotional posters in groups” and “Produce educational micro-lesson videos on diseases”. (see Fig. 1 for details).

Now, taking “Convulsion” as an example, we will elaborate on the teaching process of “One Case to the End” based on BOPPPS. The case is derived from Module 3 in Table 2, which focuses on Convulsions. (see Table 3 for details).

**Table 3:** “One case to the End” based on BOPPPS in Pediatric Nursing Teaching Process (Taking “Nursing Care for Children with Convulsions” as an example)

	**Theory Teaching (1 credit hour)**		**Experimental Teaching (3 credit hours)**	
	**Teacher Activities**	**Student Activities**	**Teacher Activities**	**Student Activities**
**Before Class**	1 week before the class, the Rain Classroom [38] pushed out the preparatory courseware on convulsions; the class QQ group released preparatory tasks and clinical case studies; and the online learning platform also promoted for students to access additional resources and further learning materials [39]	prepared courseware; thought and analyzed clinical cases; read cutting-edge literature; group case discussions	1 week before the class, the class QQ group released clinical case studies; sent videos on scalp intravenous infusion and first aid for convulsion; and assigned learning tasks involving scenario-based case drills	Checked the learning tasks; Watched videos; Group discussion to analyze cases; Role assignment and practice as required
**During** **Class:** **B**	①Started teaching via Rain Classroom; ②Introduced a clinical case; ③Presented the story of the case; ④Encouraged students to think: “What is febrile convulsion? Why are children more susceptible to it? How should febrile convulsions be treated?”; ⑤Introduced the theme of the lecture	Listened to the case story and reflected on the questions	①Started teaching via Rain Classroom; ②Introduced a clinical case; ③Posed questions: In groups, conduct scenario-based case drills and implement the correct emergency measures for Hanhan. Complete the following tasks (prepare the solution, perform scalp vein infusion, measure vital signs, and provide care for high fever); ④Guided students in thinking and discussion	Discussed the questions in groups and assigned roles according to the tasks
**During** **Class:** **O**	Stated the learning objectives: “Be able to describe the clinical manifestations, nursing diagnoses, and interventions for convulsions; swiftly assess the condition to provide timely and accurate treatment; cultivate a medical practitioner’ s ethos of caring for child patients and saving lives.”	Listened carefully and took notes	Stated the learning objectives: “Be able to memorize the precautions for measuring children’s vital signs and performing scalp intravenous infusion; safely and accurately execute the scalp vein infusion procedure; cultivate empathy and establish a caring consciousness.”	Listened carefully and took notes
**During** **Class:** **P**	①Pushed out pre-test questions: “Which of the following statements about convulsions is incorrect?……” to assess students’ self-study effectiveness; ②Provided targeted content instruction based on the students’ answers	Thought and answered the questions	①Pushed out pre-test questions: “A 3-year-old child is admitted to the hospital due to recurrent convulsions. Which of the following drugs can be used for emergency treatment?……” to assess students’ self-study effectiveness; ②Provide targeted content instruction based on the students’ answers	Thought and answered the questions
**During** **Class:** **P**	①Explained the definition of seizures; ②Analyzed the medical history and auxiliary examination datas in the case to guide students’ thinking: Discuss the cause of Hanhan's febrile seizures and provide an explanation; ③Detailed the clinical manifestations by combining Hanhan’ s sudden seizure symptoms from the case with typical images and videos of seizure episodes;④Invited students to use an infant model to demonstrate first aid for sudden seizures, followed by teacher guidance and feedback, then showed the correct first aid process and emphasized precautions; ⑤Sent questions through the Rain Classroom: What harm could incorrect first aid measures possibly bring to Hanhan?; ⑥Led a discussion with students to identify current nursing problems for Hanhan in the case, and invited students to answer how to develop targeted nursing interventions	①Listened and took notes; ②Considered the significance of abnormal test results to infer the cause of Hanhan’ s febrile seizures;③Reviewed the medical history, watched videos and viewed images of seizure episodes to deepen the understanding of the clinical manifestations of seizures;④Observed student demonstrations, discussed and analyzed the correct first aid procedures for seizures according to teacher feedback; ⑤Completed quiz questions through the Rain Classroom platform;⑥Participated in group discussions on current nursing problems, listed corresponding nursing measures from different perspectives, and shared insights	①Drew lots to determine the order of group scenario case drills; ②Watched drills from each group, invited students to self-assess and peer assess; ③Analyzed cases, pointing out key points and difficulties in operation; ④Commented on each group’s operation and demonstrated standard procedures; ⑤Emphasized humanistic care and empathy; ⑥Guided students in group practice and promptly corrected operational errors; ⑦Randomly selected students to demonstrate operations and provide comments; ⑧Summarized the experimental process and precautions	①The group leader drew lots and assigned roles within the group; ②Each group reenacted the case process in the order determined by the lot drawing; ③Self-assessment within groups, peer -assessment, and discussion between groups; ④Listened to the teacher’s case analysis and took notes; ⑤Watched teacher’ s demonstration, marking key points and difficulties; ⑥Practiced operations in groups and received guidance from the teacher;⑦Watched classmates’ demonstration of operations and provided comments; ⑧Memorized key points and difficulties, reconstructing the operation process
**During** **Class:** **P**	Pushed out post-test questions: “Hanhan’ s condition has improved and she is preparing for discharge tomorrow. Which of the following health education contents provided to her and her family is inappropriate?……” in order to promptly assess the mastery of knowledge points	Considered Hanhan's current condition and then thought and answered the questions	Pushed out post-test questions: “Which of the following actions during Hanhan’ s emergency treatment could potentially lead to accidental physical injury?……” in order to promptly assess the mastery of knowledge points	Considered Hanhan’s current condition and then thought and answered the questions
**During** **Class:** **S**	Displayed typical images of convulsion episodes and summarized the five steps of emergency first aid as “Lie down, Remove, Turn, Clear, Transport” to facilitate students’ memorization and review	Listened and marked the key points and difficulties	Summarized and reiterated the five steps of emergency first aid as “Lie down, Remove, Turn, Clear, Transport”, emphasizing the integration of humanistic care and attention to psychological nursing during the operation	Listened and marked the key points and difficulties
**After Class**	①Assigned homework in the class QQ group: “Students in groups create a home care promotional poster for Hanhan”; ②Reviewed the assignments and provide feedback	①Group discussion to think about the division of labor and production process for the promotional poster; ②Completed the assignment and submit it on time	①Assigned homework in the class QQ group: “In groups, create a science popularization video about convulsions” and upload it to the class QQ group; ②Received the assignments and graded them	①Group discussion to think about the division of labor and production process for the video; ②Completed the assignment and submitted it on time

### Evaluation metrics

Comparison of the two groups’ academic achievements, level of nurses’ professional values, and critical thinking abilities at the end of the semester after the course instruction, and investigation of the experimental group’s satisfaction evaluation of the curriculum reform.

1. Academic Achievements It consisted of final theoretical examinations, scenario case simulation and case nursing documentation and presentation of pediatric nursing. ①The final theoretical exam accounting for 50% of the total grade. The questions for both groups covered the same scope, with the same level of difficulty, and were marked and graded by the same standards and teachers, all under closed-book conditions.The total score of the test paper was 100 points, including 40 points for single-choice questions, 30 points for short-answer questions and 30 points for case analysis questions, with a duration of 120 min. Questions were randomly selected from the nurse qualification examination question bank, assessing students' grasp of fundamental knowledge points, understanding and application of common diseases, and their clinical analytical skills for various systemic diseases, as well as their ability to flexibly apply critical thinking to solve common clinical nursing issues. ②In the scenario case simulation, students worked in groups to draw clinical cases on-site for role-playing, training their abilities to conduct nursing assessments for children admitted to the hospital, handle medical orders, respond to clinical emergencies, propose and establish nursing diagnoses and measures. Through inter-group collaboration, it can enhance students' sensitivity to clinical contexts, strengthen their clinical procedural skills, improve clinical adaptability and critical thinking, reinforce humanistic care for pediatric patients, and enhance their professional identification with their work. ③Case nursing documentation and presentation involved students working in groups to write reflections on clinical cases collected during their internships in NICU or pediatric wards, and to create a PowerPoint presentation. It primarily assessed students’ literature research, writing and presentation skills. Scenario case simulation and case nursing documentation and presentation of Pediatric Nursing Case, both of which are scored according to the grading criteria by a group basis, with a total score of 100 points, accounting for 30% and 20% of the total grade, respectively.
2. The Nurse Professional Values Scale (NPVS) It was adapted and localized based on the translation and back-translation process conducted by Gong [40]. The KMO statistic of this scale is 0.966, and the overall Cronbach’s α coefficient is 0.959. The Cronbach’s α coefficients for each subscale are all greater than 0.7, indicating good reliability and validity [40]. This makes it an appropriate evaluation tool for assessing the professional values of nursing undergraduates in China [41, 42]. The scale consists of 26 items in four dimensions: care provision, professional integrity, trust and activism. Each item is rated on a 5-point scale based on its importance to the subject’s job content, ranging from “not important” (1 point) to “extremely important” (5 points). The total score of the scale ranges from 26 to 130, with higher scores indicating a more positive professional values orientation [40]. In our study, the Cronbach’s α coefficient of the scale was 0.829, and the KMO value was 0.915.
3. The Critical Thinking Scale (CTS) It was developed by Yoon in 2004, and later, Chinese scholar Yuan adapted and modified the the Chinese version of the questionnaire according to national conditions of China in 2008 [43]. The scale was consisted of 27 items in seven dimensions: truth-seeking, analytical ability, open-mindedness, systematical ability, self-confidence in critical thinking, intellectual curiosity, and cognitive level. The scale was scored on a 5-point Likert scale, with higher scores indicating greater critical thinking ability. A total scale score greater than 80 or a mean score greater than 3 indicated a positive level of critical thinking ability [43]. The overall Cronbach’s α coefficient of this scale is 0.7813, and the test–retest reliability is 0.834, indicating good internal consistency and stability [43]. It is suitable for assessing the critical thinking abilities of nursing undergraduates in our country [44]. In our study, the Cronbach’s α coefficient of the scale was 0.852, and the KMO value was 0.914.
4. Course Reform Satisfaction Evaluation It was developed by the researcher after reviewing literature, consulting with teaching faculty, and gathering opinions from students, which gauge students’ satisfaction and affection for the new teaching model. Its contents contained four questions: preferred methods of pediatric nursing instruction, satisfaction with the “One Case to the End” simulation of clinical scenarios, satisfaction with the enhancement of humanistic care and clinical thinking skills through the “One Case to the End” approach, satisfaction with the teaching effectiveness of the BOPPPS-based “One Case to the End” teaching model, etc. Each question was rated on a three-tier scale: satisfied, neutral and dissatisfied.

### Statistical analysis

In this study, a total of 276 questionnaires were distributed through the online survey platform “Wenjuanxing” [45] and all 276 were returned (validity rate 100%). After encoding the survey data, it was double-checked and entered into Excel 2013. Data analysis was analyzed using the SPSS 29.0 software, with a two-tailed test and a significance level of α = 0.05, and all of which met the normal distribution and had a chi-square variance after the normal test and ANOVA test. Mean ± standard deviation, frequency and rate were used to describe the demographic data, professional values and critical thinking scores. Independent samples t-tests were used to compare the differences in academic performance, professional values and critical thinking abilities between the students in the control group and the experimental group. Percentages were used to understand students’ satisfaction with the curricular reforms of the new teaching model.

## Results

1. Comparison of demographic data between the two groups of students
  The control group consisted of 138 individuals with an average age of (20.94±0.942) years, including 20 male students, and 138 students in the experimental group, with an average age of (20.85±0.810) years, including 22 male students. There was no statistically significant differences between the two groups in terms of other demographic datas, enrollment scores, and grades in public and foundational professional courses (*p*>0.05), indicating comparability. The two groups were compared for demographic differences in terms of ehnic group, place of origin, religious belief, serving as a class cadre, the intention to apply for nursing major, the degree of enthusiasm for the nursing major, an interesting in nursing, be in nursing profession after graduation, parents’ supportive of becoming a nurse, and showing no statistical differences (*p* > 0.05). (see Table 4 for details).

**Table 4:** Comparison of demographic data between two groups

Items	Categorization	Control Group(*n* = 138)	Experimental Group(*n* = 138)	*x*^2^	*P-value*
Ethnic Group	Han ethnic group	91 (65.94%)	83 (62.32%)	0.995	0.318
	Ethnic Minorities	47 (34.06%)	55 (39.86%)		
Place of Origin	Urban Area	34 (24.64%)	28 (20.29%)	0.749	0.387
	Rural Area	104 (75.36%)	110 (79.71%)		
Religious Belief	Presence	3 (2.17%)	2 (1.45%)	0.204	0.652
	Absence	135 (97.83%)	136 (98.55%)		
Whether to serve as a class cadre or not	Yes	30 (21.74%)	23 (16.67%)	1.144	0.285
	No	108 (78.26%)	115 (83.33%)		
The intention to apply for the nursing major	Voluntary	94 (68.12%)	89 (64.50%)	3.671	0.160
	Family's wishes	28 (20.29%)	22 (15.94%)		
	Major reassignment	16 (11.59%)	27 (19.57%)		
The degree of enthusiasm for the nursing major	Very much like	4 (2.90%)	5 (3.62%)	4.928	0.295
	Quite like	65 (47.10%)	54 (39.13%)		
	Uncertain	58 (42.03%)	62 (44.93%)		
	Dislike	8 (5.80%)	16 (11.59%)		
	Hate	3 (2.17%)	1 (0.72%)		
Is there an interest in nursing	Yes	106 (76.81%)	94 (68.12%)	2.615	0.615
	No	32 (23.19%)	44 (31.88%)		
Whether or not you will be in the nursing profession after graduation	Yes	71 (51.45%)	67 (48.55%)	2.234	0.525
	Possibly	66 (47.83%)	70 (50.72%)		
	Haven’t considered it yet	0 (0)	1 (0.72%)		
	No	1 (0.72%)	0 (0)		
Were your parents supportive of you becoming a nurse	Approve	112 (81.16%)	102 (73.91%)	2.152	0.341
	Indifferent	23 (16.67%)	31 (22.46%)		
	Disapprove	3 (2.17%)	5 (3.62%)		

Control group: control group with traditional teaching methods

Experimental Group: experimental group with “One Case to the End” teaching model based on BOPPPS method. The two groups were compared using the chi-square test

2. Comparison of academic achievements between two groups of students
  By independent samples t-test, there was a significant difference between the control group and the experimental group in final theoretical exam scores, scenario case simulation scores, and case nursing documentation and presentation scores (*p* < 0.05), and all scores of the experimental group were significantly higher than those of the control group (see Table 5 for details).

**Table 5:** Comparison of academic achievements between two groups (M ± SD)

Item	Control Group(n = 138)	Experimental Group(n = 138)	*t*	*p-value*
Final Theoretical Exam	69.65 ± 4.49	74.04 ± 6.75	-6.36	< 0.01
Scenario Case Simulation Scores	77.28 ± 4.43	80.15 ± 6.47	-4.30	< 0.01
Case Nursing Documentation and Presentation Scores	83.81 ± 2.96	87.55 ± 1.93	-12.45	< 0.01

The two groups were compared using the independent-samples t-test

*Abbreviations:* *M* mean values, *SD* standard deviation

3. Comparison of professional value competencies between two groups of students
  Through the independent samples t-test, the total score of the experimental group’s professional value competence and the scores of each dimension were higher than those of the control group, and the difference was significant (*p* < 0.05) (see Table 6 for details).

**Table 6:** Comparison of professional values competencies between two groups (M ± SD)

Item	Item number	Control Group(*n* = 138)	Experimental Group(*n* = 138)	*t*	*p-*value
Care Provision	10	38.95 ± 1.71	43.27 ± 1.54	-22.07	< 0.01
professional integrity	7	25.87 ± 1.23	28.53 ± 1.34	-17.20	< 0.01
Trust	6	18.09 ± 1.13	21.53 ± 1.24	-24.11	< 0.01
Activism	3	11.96 ± 0.80	12.93 ± 0.80	-10.10	< 0.01
Total Score	26	94.86 ± 2.76	106.25 ± 2.45	-36.29	< 0.01

The two groups were compared using the independent-samples t-test

4. Comparison of critical thinking abilities between two groups of students
  An independent samples t-test was conducted to compare the critical thinking abilities between two groups of students. The results showed that the experimental group scored significantly higher than the control group in critical thinking ability total score as well as in the score of each dimension, with a statistically significant difference (*p*<0.05) (see Table 7 for details).

**Table 7:** Comparison of critical thinking abilities between two groups (M ± SD)

Item	Item number	Control Group	Experimental Group	*t*	*p-value*
Truth-Seeking	4	10.11 ± 1.02	12.48 ± 0.93	-20.14	< 0.01
Analytical ability	4	15.18 ± 0.81	16.04 ± 0.82	-8.68	< 0.01
Open-Mindedness	4	14.78 ± 0.79	15.82 ± 0.82	-10.74	< 0.01
Systematical Ability	4	14.99 ± 0.88	17.09 ± 0.97	-18.75	< 0.01
Self-Confidence in Critical Thinking	4	14.45 ± 1.00	15.70 ± 0.92	-10.81	< 0.01
Intellectual Curiosity	4	15.17 ± 0.96	17.46 ± 0.83	-21.32	< 0.01
Cognitive Level	3	9.00 ± 0.82	12.28 ± 0.72	-35.25	< 0.01
Total Score	27	93.67 ± 2.46	106.86 ± 2.50	-44.23	< 0.01

The two groups were compared using the independent-samples t-test

**Fig. 2:**
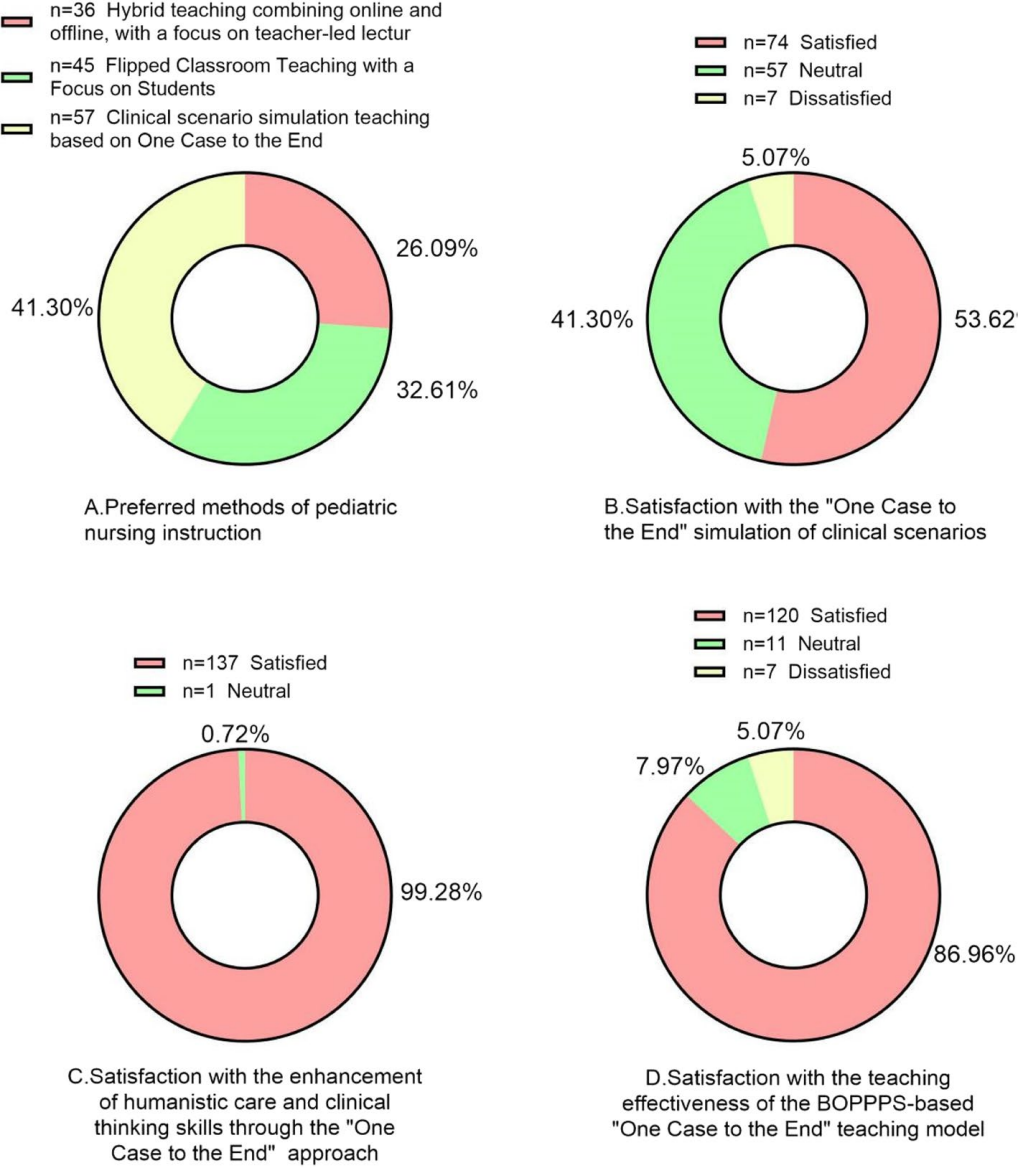
Satisfaction evaluation of curriculum reform by students in the experimental group (*n* = 138)

5. Satisfaction evaluation of curriculum reform by students in the experimental group
  A satisfaction evaluation survey on pediatric nursing curriculum reform was distributed to 138 students in the experimental group. The results showed that more than 40% of the students currently prefer the “One Case to the End” throughout the teaching process. A total of 53.6% of the students were satisfied with the “One Case to the End” simulation of clinical scenarios. A staggering 99.3% of the students believed that the “One Case to the End” method could enhance humanistic care literacy and clinical thinking skills. Furthermore, 87% of the students were satisfied with the teaching effectiveness of the “One Case to the End” teaching model based on BOPPPS (see Figure 2 for details).

## Discussion

This study implemented “One Case to the End” teaching model based on BOPPPS in the theoretical and experimental teaching of Pediatric Nursing to explore its impact on the learning outcomes of undergraduate nursing students. This innovative teaching model helped to improve nursing students’ academic performance, cultivate their professional values and critical thinking abilities, leading a high recognition by the students of the reform achievements in Pediatric Nursing.

### Improving academic performance of nursing students

Nursing students who adopted the “One Case to the End” teaching model based on BOPPPS achieved higher scores in their final exams, scenario case drills, case nursing presentation and writing compared to those who used traditional teaching methods (*p* < 0.001). This indicated that the “One Case to the End” teaching model based on BOPPPS can enhance students’ mastery of theoretical knowledge, improve their clinical operational skills, and strengthen their abilities in medical history taking and medical record writing.

In this study, learning tasks were released one week before the class, providing students with ample time to review and preview relevant materials, thus promoting active learning [46, 47]. Additionally, students sometimes needed to discuss clinical cases with peers, assign scenario roles, which also helped to enhance their spirit of teamwork and collaboration [48]. During the class, the instructor introduced clinical cases that run consistently through the six phases of BOPPPS. By following the progression of the case, the teaching dynamics were kept up-to-date, with a series of educational activities such as discussions, quizzes, scenario role-playing, case analysis, and practical operations being set up. Through effective guidance from the instructor, this approach captured students’ attention, stimulated their interests and initiatives in learning [49], enhanced practical skills, and deepened their understanding of theoretical knowledge as well as classroom engagement. This contributed to improved academic performance among nursing students [50]. After class, the clinical cases were extended into daily life by having groups of students create health education and science popularization materials related to diseases. This not only helped them review and master theoretical knowledge but also enhanced their ability to practically apply that knowledge. Both BOPPPS and the “One Case to the End” were student-centered teaching strategies [23, 33]. By using the “One Case to the End” teaching model based on BOPPPS, traditional “Didactic Teaching” instruction was transformed into a collaborative teaching and learning activity involving both teachers and students [22]. This provided nursing students with more opportunities for hands-on practice, skill development and peer learning, thereby effectively enhancing learning efficiency.

### Fostering professional values in nursing students

The professional values of nurses were the cornerstone for enhancing the quality of care [51].

They represented the enduring beliefs and principles that nurses hold regarding the characteristics and evaluation of the nursing profession [52]. These values can influence an individual’s professional behavior and practice decision [53], thereby guiding action including those in nursing management [54]. Additionally, nurses with positive values can provide more humanized care to patients [55], which is also a guarantee of nursing quality [56]. The results of this study showed that the total score of students’ professional value competence in the experimental group, as well as the scores in four dimensions including care provision, professional integrity, trust and activism were all higher than those in the control group (*p* < 0.001), which indicated that the “One Case to the End” based on BOPPPS teaching model was effective in cultivating students’ levels of professional values.

“One Case to the End” based on BOPPPS teaching model, during theoretical lectures, instructors incorporated positive news events or stories of exemplary figures in the profession related to the teaching content. These were used in conjunction with clinical cases, which not only expanded teaching resources and enriched the curriculum but also attracted students’ attention in class. This subtle approach enhanced students’ sense of professional mission and responsibility [57], guiding them to develop positive and upward-looking professional values [58]. In the experimental class, clinical cases were used throughout to create a clinical context, integrating components of humanistic care, nurse-patient communication, ethics, and emergency symptom management [26]. Through scenario role-playing, students were placed in realistic situations that recreate the progression of the case. This allowed them to experience the perspectives and emotions of nurses, pediatric patients, or their families from different roles and mindsets. Through group collaborative scenario simulations, the core competencies of nursing students were effectively enhanced [59, 60], which in turn improved their professional nursing behaviors. This prepared them to provide high-quality, humanized care to pediatric patients and their families.

### Enhancing critical thinking skills in nursing students

Cultivating students’ critical thinking skills, with a particular emphasis on innovative and evaluative thinking, was a crucial aspect of higher nursing education [61]. Critical thinking skills can enhance nurses’ evidence-based nursing capabilities, enabling them to use effective clinical judgments and decision-making skills to improve patient prognosis, thereby providing professional, safe and high-quality care to patients [9]. In this study, it showed that the scores of the experimental group were higher than those of the control group in the dimensions of total critical thinking ability and its seven dimensions (*p* < 0.001), indicating that “One Case to the End” based on BOPPPS teaching model can enhance students’ critical thinking abilities.

In the “One Case to the End” based on BOPPPS teaching model, as clinical cases progressed and questions became more complex, the teacher’s patient guidance and active encouragement can continuously stimulate students’ curiosity and enhance their engagement in learning [62, 63]. Engaging in classroom discussions and sharing experiences can help students find the courage to express themselves and build their self-confidence [64]. By participating in scenario case drills as part of a group, students can learn to calmly manage complex and rapidly changing emergency situations within clinical settings involving pediatric patients. These exercises trained their comprehensive thinking and appropriate handling of clinical nursing issues, emphasizing clinical judgment, communication skills, critical thinking, and clinical decision-making [65, 66]. This ultimately enhanced their ability to analyze and resolve problems. Nursing students, as the future custodians of healthcare, need to have robust critical thinking abilities to protect the lives and safety of their patients.

The “One Case to the End” based on BOPPPS teaching model, prioritized a “student-centered” philosophy [67].This approach encouraged students to take an active role in their learning, construct their own knowledge frameworks, and ultimately enhance their critical thinking abilities.

### Students satisfied with the outcomes of teaching reform

Students expressed satisfaction with the innovative teaching model of “One Case to the End” based on BOPPPS teaching model implemented in the Pediatric Nursing course. Especially in the “One Case to the End” simulation of clinical scenarios, students’ enhancement of humanistic care and clinical thinking skills through the “One Case to the Case” approach received high praise from more than half of the students. The satisfaction level with the teaching effectiveness of the BOPPPS -based “One Case to the End” teaching model was nearly 90%. However, regarding the teaching methods in pediatric nursing, only about 41% of the students indicated that they liked the clinical scenarios simulation teaching based on “One Case to the Case”. In contrast, approximately 33% and 26% of the students preferred the flipped classroom teaching with a focus on students, and hybrid teaching combining online and offline, with a focus on teacher-led lecture, respectively. The reason for this may be that the “One Case to the End” teaching model based on BOPPPS, which spanned from preparation to implementation and completion, covered nearly half a month and required the full participation of both teachers and students throughout this period. Teachers may not be able to fully supervise the process due to personal reasons [68], or students might be accustomed to traditional passive teaching methods, leading to maladjustment in learning [65]. This can result in an inability to complete learning tasks effectively, thereby affecting their regular grades, which inevitably leads to dissatisfaction with the new teaching model. Additionally, some learning tasks involved self-directed inquiry and team collaboration, which made some students feel that the workload was heavy and difficult to manage [69]. This can lead to procrastination or hinder the efficiency of group work, impacting team relationships. This suggested that teachers should appropriately shorten the duration of teaching spans, focusing on concentrating students’ tasks within classroom instruction, increasing student-centered classroom activities, and reducing the learning burden before and after class. When assigning course tasks and arranging group collaborations, we should take into consideration the varying learning abilities and autonomy levels of students at different levels [70, 71]. It was advisable to control the number of students in each group and implemented a combination of “high-performing, good, and average” students to avoid free grouping among students. This approach maximized the opportunity for each student to meet more new friends, thereby expanding their social network, and encouraged them to fully utilize their individual roles within the group. They should actively participate in case scenario drills, role-playing, and discussion analysis processes. Meanwhile, teachers should also provide appropriate encouragement measures. For instance, for students with average grades, teachers could give a slight advantage in their regular scores based on their classroom performance or completion of pre-class tasks. It was also important to promptly collect students’ feedback on innovative teaching reforms and made adjustments and improvements accordingly.

Notably, Coşkun et al. [72] conducted a quasi-experimental study in 2024 evaluating the impact of structured “Family-Centered Care” (FCC) training on the attitudes of pediatric nurses. The results confirmed the hypothesis that “FCC training has a positive impact on the attitudes of pediatric nurses.” Coşkun et al. adopted the Yale method, emphasizing innovative educational technologies such as multimedia and gamification. This approach aligned well with our study’s use of a clinical case-based throughout teaching method within the BOPPPS model. Both methods share a common goal of fostering modern teaching frameworks, aiming to enhance educational outcomes through structured, innovative cultivation approaches. These strategies are designed to promote active learning and attitude changes among healthcare professionals, thereby strengthening educational practices. Through comparative analysis, it is evident that both methods can effectively enhance educational quality in different settings. Coşkun et al.’ s research provided additional evidence for the effectiveness of modern educational frameworks within healthcare settings. Their findings on the sustained impact of FCC (Family-Centered Care) training further support our argument that structured training models, such as the “One Case to the End” teaching model based on BOPPPS, can generate lasting benefits in contemporary nursing education. This indicates that such structured approaches have broad applicability that extends beyond specific environments.

### Limitations

This study also has the following limitations. First, the population surveyed was limited to the College of Nursing, and the study population and sample size could be expanded to comprehensively assess the effectiveness of this teaching model and generalize the results further. Second, the use of a single survey method in this study was not conducive to an in-depth understanding of the impact of the model on the psychological dimensions of the students’, which may limit the interpretability of the results. Subsequent study should be followed up by doing faculty-student interviews in conjunction with the qualitative research methodology in order to assess the continued validity of the method. Third, due to time constraints, some long-term effects were not captured in a timely manner, and a longitudinal study across several academic years is needed to reveal long-term effects over time. Fourth, due to individual differences and the complexity of the teaching environment, it was challenging to ensure that every teacher and student fully adhered to the prescribed requirements during the implementation of this study. This potential non-adherence may have affected the accuracy of the study results and the actual effectiveness of the intervention. Future research should further explore more effective monitoring and incentive mechanisms to enhance teachers’ and students’ engagement and compliance, thereby allowing for a more accurate assessment of the intervention’ s impact.

## Conclusion

In this study, we implemented a “One Case to the End” teaching model based on BOPPPS in the theoretical and experimental Pediatrics Nursing curriculum. Our findings indicated that this innovative teaching model not only improved students’ academic performance but also helped cultivate good professional values and sharp critical thinking skills. Although this study was conducted in a single environment, our findings provided valuable insights and empirical evidence for the application of innovative teaching models in Pediatric Nursing education. The innovative application of the “One Case to the End” teaching model within the BOPPPS framework is a new approach to enhancing student engagement and improving learning outcomes. It was also a novel teaching strategy applicable to various educational settings worldwide, aligning with the global trend of active learning and student-centered education. Therefore, our study offered a scalable and adaptable solution that can be implemented in different educational contexts to meet the unique needs of diverse student populations and healthcare systems. Our research not only contributed to the growing discussion on innovative teaching methods in nursing education but also highlighted the potential for broader application across different nursing specialties. This study inspired educators to explore more student-centered interactive teaching methods and encouraged the adoption of evidence-based teaching practices, thereby promoting students’ in-depth understanding of clinical reasoning and patient care. For future research, we will focus on longitudinal studies to expand its applicability and explore the possibilities of using this model in different educational environments, ensuring its broad effectiveness and adaptability.

## Supplementary Information

Supplementary Material 1.

## Data Availability

The datasets used and/or analyzed during the current study are available from the corresponding author on reasonable request.

## References

[1] Cui Y, Zhang YX. Pediatric Nursing (Seventh Edition). Beijing: People’ s Medical Publishing House; 2021.

[2] Aslı AK, Dijle A, Hamide Z. Therapeutic communication skills level among students undertaking the pediatric nursing course and the associated influencing factors. J Pediatr Nurs. 2023;73:34–43. 37603925 10.1016/j.pedn.2023.08.015

[3] OseiAppiah E, Appiah S, Kontoh S, Mensah S, Awuah DB, Menlah A, Baidoo M. Pediatric nurse-patient communication practices at Pentecost hospital, Madina: A qualitative study. Int J Nurs Sci. 2022;9(4):481–9. 36285089 10.1016/j.ijnss.2022.09.009 PMC9587402

[4] Wang W. The effect of quality nursing care in pediatric care. Clinical Medicine Frontiers. 2022;2(2):54–6.

[5] Lu QM, LY, Li YJ, Li SN, Luo YN, Huang XP, Huang ZR. Problem-oriented pedagogy in the pediatric nursing classroom. Chi Youjiang J. 2024;52(2):190–2.

[6] Chen XR. Research on the effect of applying all-round care in the nursing work of pediatric intensive care unit. International Journal of Nursing Research. 2020;2(2):103–5.

[7] Wang XJ, Jiang H, Xiao SQ, Fan L. Design and preliminary application of a newborn PICC catheterization operation virtual simulation training assessment system. Chin J Nurs Educ. 2022;19(01):10–4.

[8] Yu BY, Zeng H, Bu LJ. Effects of occupational values and achievement motivation on organization citizenship behavior and job burnout of pediatric emergency nurses in grade-A tetiary hospitals of Shanghai. Occup Health. 2020;36(03):329–33+338.

[9] Li YH, Li R, Jiang WH, Yang XQ, Yao D, Li JM, Li X, Zhang R. Study on the status and influcing factors of critical thinking ability of pediatric nurses. J Nurs Adm. 2023;23(7):566–9.

[10] Banta-Wright SA, Wright BM, Taha AA, Miehl N. Branching Path Simulation for Pediatric Nurse Practitioner Students to Promote Critical Thinking: A Quasi-Experimental Study. J Pediatr Health Care. 2024;38(5):737–46. 38661592 10.1016/j.pedhc.2024.03.004

[11] Ayed A, Ejheisheh MA, Salameh B, Batran A, Obeyat A, Melhem R, Alkhatib S. Insights into the relationship between professional values and caring behavior among nurses in neonatal intensive care units. BMC Nurs. 2024;23(1):692. 39334248 10.1186/s12912-024-02343-8 PMC11437965

[12] Horsfall J, Cleary M, Hunt GE. Developing a pedagogy for nursing teaching learning. Nurse Educ Today. 2012;32(8):930–3. 22100421 10.1016/j.nedt.2011.10.022

[13] Monly Man-Yee Y, Engle Angela C, Miranda Man-Ying W, et al. Teaching and learning strategies foster the development of autonomous learning and its interrelated factors in prelicensure nursing education: A scoping review. Teaching and Learning in Nursing. 2024;19(2):e288–97.

[14] Hu K, Ma RJ, Ma C, et al. Comparison of the BOPPPS model and traditional instructional approaches in thoracic surgery education. BMC Med Educ. 2022;22(1):447. 35681190 10.1186/s12909-022-03526-0 PMC9185859

[15] Li ZY, Cai XY, Zhou KB, Qin JY, Zhang JH, Yang QH, Yan FX. Effects of BOPPPS combined with TBL in surgical nursing for nursing undergraduates: a mixed-method study. BMC Nurs. 2023;22(1):133. 37088853 10.1186/s12912-023-01281-1 PMC10122814

[16] Padilha JM, Machado PP, Ribeiro A, et al. Clinical virtual simulation in nursing education: randomized controlled trial. J Med Internet Res. 2019;21(3): e11529. 30882355 10.2196/11529 PMC6447149

[17] Xu X, Zhang JM, Zhou YY, Wu QP, Ling H. Application of BOPPPS combined with senario simulation method in clinical nursing teaching of thoracic surgery with integrated ideology and politics theories teaching in all courses. Nursing Practice and Research. 2024;21(10):1455–61.

[18] Li J, Feng XQ, Ren WJ, Wei XH. The impact combining problem-based learning with traditional classroom-based delivery model on the critica thinking of pediatric nursing students. Shanxi Medical Journal. 2019;48(24):3120–1.

[19] Yang YY, Chen H, Sun HY. Nursing Undergraduate Students’ Experiences and Perceptions of Blended Learning in Pediatric Nursing: A Mixed Methods Study. SAGE Open Nurs. 2024;10:1–11. 10.1177/23779608241274214 PMC11384974 39258221

[20] Yang HP, Shi MF, Quan Y, Lin LP, Du M. Research on Pediatric Nursing Teaching Strategies Based on Talent Training. Chinese General Practice. 2021;24(S2):217–9.

[21] Zhang H, Gao HX, Dong YF. Practice of problem-based learning using mobile devices in undergraduate pediatric nursing education. J Nurs Sci. 2018;33(09):73–6.

[22] Chu J, Hu Q, Xu J, Wan YL, Yan M, Guo HL, Yang CX, Wang YQ. Effects of intergrating BOPPPSA into online/offline blended learning in teaching caring-oriented technical and procedural skills for nursing students. J Nurs Sci. 2024;29(17):89–92.

[23] Li F, Wang TT, Wang L, W HH, Yan P. Application of blended teaching based on BOPPPS teaching mode on undergraduate nursing teaching. Chin Nurs Res. 2023;37(22):4126–28.

[24] Li P, Lan XP, Ren L, Xie XP, Xie HT, Liu SQ. Research and practice of the BOPPPS teaching model based on the OBE concept in clinical basic laboratory experiment teaching. BMC Med Educ. 2023;23(1):882. 37978370 10.1186/s12909-023-04822-z PMC10657023

[25] Yang LQ, Chen LY, Lin ZQ, Wu QQ, Zhu CY, Zhu LM. Application of innovative high simulation scenario simulation teaching integrated integrated into nursing risk education model in comprehensive experimental teaching of nursing. Chin Nurs Res. 2019;33(13):2306–10.

[26] Feng Y, He CY, Ding X, Yang Q, Chen XJ, Wang YY. Application of BOPPPS combined with scenario simulation in teaching or Medical Nursing. J Nurs Sci. 2021;36(19):80–4.

[27] Chen YL, Li L, Li SY, Zhang SL, Liu H. Application of BOPPPS Model in Standardized Training for Nurses in Operating Room. J Nurs (China). 2022;29(14):18–20.

[28] Li Y, Li X, Liu Y, Li Y. Application effect of BOPPPS teaching model on fundamentals of nursing education: a meta-analysis of randomized controlled studie. Front Med (La.usanne). 2024;11:1319711. 10.3389/fmed.2024.1319711 PMC11111886 38784229

[29] Wen HL, Xu WT, Chen FL, Jiang XY, Zhang R, Zeng JH, Peng L, Chen Y. Application of the BOPPPS-CBL model in electrocardiogram teaching for nursing students: a randomized comparison. BMC Med Edu. 2023;23(1):987. 10.1186/s12909-023-04983-x PMC10740289 38129836

[30] Yao X. Hign school ideological and political lessons “One case to the end” Research on the application of issue-based teaching. Master’s thesis. Guiyang: Guizhou Normal University; 2024. p. 10.

[31] Zhang M. A case to the end: An Effective Way to Cultivate Core Literacy in Disciplinary. The Teaching of Thought and Political Study. 2018;03:25–7.

[32] Garrison RD. Online community of inquiry review: Social, cognitive, and teaching presence issues. Journal of Asynchronous Learning Networks. 2019;16(4):61–72.

[33] He SL. Research on the Application of the “One Case to the End” Teaching Method in High School Ideological and Political Education. Basic Educ Res. 2023;20:70–2+76.

[34] JanPhilipp B, Gregor B, Helen HHD, et al. Towards an integrated case method in management Education-Developing an ecosystem-based research and learning journey for flipped classrooms. Administrative Sciences. 2021;11(4):1–13.

[35] Cao J. An Application of BOPPPS Teaching Model in Grammar Teaching in Senior High School. Yan’an: Master’s thesis, Yan’an University; 2024. p. 16.

[36] Zhu LN, Yao AC, Zhang CC, Zhan YF. Application of “5G+smart classroom” based on BOPPPS model in clinical teaching of thoracic surgery nursing. J Nurs Sci. 2024;39(05):14–7.

[37] Rong SZ, Shi DJ, Liu FH, Ma HK, Zou LN. The fusion point of the joint application of PAD classroom and BOPPPS teaching mode in the training of medical students. Modern Preventive Medicine. 2024;51(11):2108–12.

[38] Rain Classroom. https://www.yuketang.cn/web. Acessed 29 October 2024.

[39] Chinese University MOOC. https://www.icourse163.org/learn/XMU-1002575005?tid=1471002504#/learn/announce. Acessed 29 October 2024.

[40] Gong YY, Wang HZ, Gao YL. Compilation and evaluation of nurse professional values scale. Chin Nurs Res. 2011;25(28):2628–30.

[41] Gong YY, Wang HZ, Gao YL. Study on relationship between the will of occupational choices and the professional value of undergraduate nursing students. Chin Nurs Res. 2011;25(18):1607–9.

[42] Gong YY, Wang HZ, Gao YL. Influencing Factors of Career Decision-Making of Nursing Undergraduate in Guangzhou. J Nurs (China). 2011;18(09):16–9.

[43] Yuan QH. The Relationship among Critical Thinking, Communication Ability and Perceived Self-efficacy in Nursing Students. Jinan: Master’s thesis, Shandong University; 2008. p. 13.

[44] Yuan QH, Lei XL, Gao JJ, Fan XZ. Relationships Among Academic Self-efficacy, Achievement Motives and Autonomous Learning Ability in Nursing Students. J Nurs Sci. 2008;3:48–51.

[45] Wenjuanxing. https://www.wjx.cn/?source=bing&plan=%E9%97%AE%E5%8D%B7%E6%98%9F&keyword=%E9%97%AE%E5%8D%B7%E6%98%9F&msclkid=addc1fd65d581a2a0e2478f6bd2b81ad. Acessed 29 October 2024.

[46] Erenel AS, Sözbir SY, Aksoy MU, Gürcüoğlu EA, Aksu SP, Toprak FÜ, Asalioğlu CU. Effect of scenario-based simulation training on the obstetrics and gynecology nursing clinical practicum. J Nurs Res. 2021;29(2):e142. 33395173 10.1097/jnr.0000000000000417

[47] Patelarou AE, Mechili EA, Ruzafa-Martinez M, Dolezel J, Gotlib J, Skela-Savič B, Ramos-Morcillo AJ, Finotto S, Jarosova D, Smodiš M, Mecugni D, Panczyk M, Patelarou E. Educational interventions for teaching evidence-based practice to undergraduate nursing students:a scoping review. Int J Environ Res Public Health. 2020;17(17):6351. 32878256 10.3390/ijerph17176351 PMC7503534

[48] Peng WS, Wang L, Zhang H, Zhang Z, Wu YM, Sang X, Zhou R, Xu JL, Chen X. Application of virtual scenario simulation combined with problem-based learning for paediatric medical students. J Int Med Res. 2021;49(2):1–7. 10.1177/0300060520979210 PMC7871091 33554701

[49] Zhang YW. The reform path of student-centered teaching mode in secondary nursing profession education. Asia-Pacific Education. 2024;10:176–8.

[50] Feng RH. Application of BOPPPS integrated scenario simulation teaching method in the teaching of geriatric nursing in Secondary vocational schools. Qingdao: Master’s thesis, Qingdao Universitya; 2022. p. 26.

[51] Büşra E, Gülşah GA, Cahide A, Dilek Ö. The effects of an ethics laboratory program on moral sensitivity and professional values in nursing students: a randomized controlled study. Nurse Educ Today. 2022;111:105290. 35144203 10.1016/j.nedt.2022.105290

[52] Chen SR. The Current Status and Correlative Factors of Humanistic Care Ability in the Standardized Training Nurses. Zhengzhou: Master’ s thesis, Henan University; 2019. p. 3.

[53] Su XD, Miao KK, Chen Q. Effect of moral sensitivity on professional values of undergraduate nursing students: Mediating effect of empathic tendency. Med Soc. 2021;34(05):105–10.

[54] Podgorica N, Rached CDA, Crescente NY, et al. Nursing Professional Values Scale (NPVS-3) in an Austrian context: validation of a scale and reliability assessment. BMC Nurs. 2024;23(1):510. 39075433 10.1186/s12912-024-02175-6 PMC11288009

[55] Cui LY. The research on Humanistic Caring Ability and Professional Values among. Dalian: Master’ s thesis, Dalian Medical University; 2016. p. 7.

[56] Kaya A, Dalgiç AI. It is possible to develop the professional values of nurses. Nurs Ethics. 2021;28(4):515–28. 33047657 10.1177/0969733020952135

[57] Xu LL, Nie XF, Li Y, Ke L, Sun L, Cao QY, Xiao J, Luo YX, Tao LX. Experimental teaching effect of Fundamentals of Nursing with full integration of soft and hard skills in the perspective of ideological and political theory education. J Nurs Sci. 2025;40(02):1–4.

[58] Xiao XJ, Zhao XF. Construction and practice of the “three forgings and five integrations” ideological and political teaching model of pediatric nursing. Chinese Evidence-Based Nursing. 2024;10(21):3909–14.

[59] Day L, Beard KV. Meaningful inclusion of diverse voices: the case for culturally responsive teaching in nursing education. J Prof Nurs. 2019;35(4):277–81. 31345507 10.1016/j.profnurs.2019.01.002

[60] Xue W, Liu GL, Liu Y. Application of TBL Combined with Scenario Simulation in Emergency Skills Training for Dental Clinic Nurses. J Qilu Nurs. 2022;28(07):165–7.

[61] Peng MC, Wang GC, Chen JL, Chen MH, Bai HH, Li SG, Li JP, Cai YF, Wang JQ, Yin L. Validity and Reliability of the Chinese Critical Thinking Disposition Inventory. Chin J Nurs. 2004;39(9):7–10.

[62] Li SZ, Wei W, Li XF, Ma L, Li QJ, Sun XiC, Chen X. Impacts of blended learning with BOPPPS model on Chinese medical undergraduate students: a comprehensive systematic review and meta-analysis of 44 studies. BMC Med Educ. 2024;24(1):914. 10.1186/s12909-024-05917-x PMC11344447 39180016

[63] Zeng XZ. Example of the implementation strategy of “One Case to the End” teaching methodology. Teaching Reference of Middle School Politics. 2020;05:39–40.

[64] Cook TC, Camp-Spivey LJ. Innovative Teaching Strategies Using Simulation for Pediatric Nursing Clinical Education During the Pandemic: A Case Study. Acad Med. 2022;97(3S):S23–7. 10.1097/ACM.0000000000004538 PMC8855774 34817401

[65] Chen X, Wang L, Zhai X, Li Y. Exploring the Effects of Argument Map-Supported Online Group Debate Activities on College Students’ critical thinking. Front Psychol. 2022;13:1–12. 10.3389/fpsyg.2022.856462 PMC9162080 35664173

[66] Jin YM, Hu Y, Zhang HY, Li YF, Tu YF, Kong NNa,Wang J. Practice and consideration for the model of student-centered teaching rounds in pediatrics. Chinese J Nurs. 2015;50(02):229–33.

[67] Chen L, Tang XJ, Chen XK, Ke N, Liu Q. Effect of the BOPPPS model combined with case-based learning versus lecture-based learning on ophthalmology education for five-year paediatric undergraduates in Southwest China. BMC Med Educ. 2022;22(1):1–7. 10.1186/s12909-022-03514-4 PMC9170341 35668389

[68] Fu H, Liu JF, Feng J, Chen XJ, He GQ, Wang YH . Accurate Supervision of Teaching in Universities under Modern Information Technology. Modern Education Forum. 2022;5(2): 1–3.

[69] Chen WX, Sun C, Li J , Niu LJ. To Compare the Application Value of High Simulation Severe Trauma Treatment Exercise and Traditional Teaching Method in Clinical Teaching of Emergency Trauma. China Health Industry. 2024;21(10):192–4.

[70] Liu J. How Does Ability Grouping Affect Students’ Math Achievement? An Empirical Study Based on PISA 2022 Data in East Asia. J Sh Educ Res. 2024;08:11–8.

[71] Liu MF, Liu WH. How Ability Grouping Instruction Promotes Students’ Individual Development. Mod Distance Educ Res. 2023;35(01):37–48.

[72] Coşkun AB, Al-Motlaq M, Pişkin M, Elmaoğlu E, Çelebioğlu A. Evaluation of the impact of family-centered care training on pediatric nurses' attitudes. J Pediatri Nurs. 2025;80:e136–43. 10.1016/j.pedn.2024.12.003 39674702

