# Supplementary material for: The application effectiveness of the “One Case to the End” teaching model based on BOPPPS in pediatric nursing: a quasi-experimental study
Source: BMC Med Educ. 2025 Apr 17;25:556. doi: 10.1186/s12909-025-07073-2 (PMC12004747; doi:10.1186/s12909-025-07073-2)
Supplement: Supplementary file 1 — Supplementary Material 1. [file 12909_2025_7073_MOESM1_ESM.doc]

**1.Demographic Questionnaire**

1. Age : years old
2. Gender: ①Male ②Female
3. Ethnic Group: ①Han ethnic group ②Ethnic Minorities
4. Place of Origin: ①Urban Area ②Rural Area
5. Religious Belief: ①Presence ②Absence
6. Whether to serve as a class cadre or not: ①Yes ②No
7. The intention to apply for the nursing major:

① Voluntary ②Family's wishes ③ Major reassignment

1. Is there an interest in nursing: ①Yes ②No

**4. Satisfaction Evaluation of Curriculum Reform by students in the Experimental Group**

1. Preferred methods of pediatric nursing instruction

①Hybrid teaching combining online and offline, with a focus on teacher-led lectures

②Flipped Classroom Teaching with a Focus on Students

③Clinical scenario simulation teaching based on "One Case to the End"

1. Satisfaction with the "One Case to the End" simulation of clinical scenarios

①Satisfied ②Neutral ③Dissatisfied

1. Satisfaction with the enhancement of humanistic care and clinical thinking skills through the "One Case to the End" approach

①Satisfied ②Neutral ③Dissatisfied

1. Satisfaction with the teaching effectiveness of the BOPPPS-based "One Case to the End" teaching model

①Satisfied ②Neutral ③Dissatisfied
